# Network characteristics of the youth’s insomnia and emotional symptoms and their gender differences

**DOI:** 10.3389/fpsyt.2025.1597652

**Published:** 2025-06-16

**Authors:** Chang Liu, Lie Zhou, Xiao-Xia Pi, Bo Liu, Xin-Feng Zhang, Wen-Can Wei, Suo-Cheng Nie

**Affiliations:** ^1^ Mental Health Center of Yangtze University, Jingzhou, Hubei, China; ^2^ Mental Health Institute of Yangtze University, Jingzhou, Hubei, China; ^3^ Department of Psychiatry, Jingzhou Rongjun Special Care Hospital, Jingzhou, Hubei, China; ^4^ Department of Psychiatry, Jingzhou Mental Health Center, Jingzhou, Hubei, China; ^5^ Mental Health Center, West China Hospital, Sichuan University, Chengdu, Sichuan, China; ^6^ Institute of Psychiatry, West China Hospital, Sichuan University, Chengdu, Sichuan, China; ^7^ Department of Psychiatry, Pingtan Comprehensive Experimental Area Hospital, Pingtan, Fujian, China

**Keywords:** insomnia, depression, anxiety, gender differences, network analysis, youth

## Abstract

**Objective:**

To explore the association between sleep disorders and symptoms of depression and anxiety in the youth and to analyze the influence of gender factors.

**Methods:**

Using the Mental Health Status Survey Questionnaire for Adolescent Students compiled by Professor Maosheng Ran, a survey was conducted and 7247 valid responses were collected (valid response rate of 79.11%). Integrating the Insomnia Severity Index(ISI), Patient Health Questionnaire(PHQ-9), and Generalized Anxiety Disorder Scale(GAD-7), network analysis was employed to assess the network structure, symptom associations, and gender differences related to insomnia, depression, and anxiety among youth.

**Results:**

In the network of insomnia, depression, and anxiety symptoms among youth, the highest strength centrality values were observed for “excessive worry,” “fatigue,” “sleep dissatisfaction,” and “distress caused by sleep difficulties.” Five bridge symptoms were identified: “fatigue,” “nervousness,” “suicidal ideation,” “motor,” and “guilt.” Significant differences in network structures existed between genders, specifically in network invariance (M = 0.909, p = 0.025) and global strength (males = 75.155, females = 70.527; S = 4.628, p = 0.041). Additionally, males showed significantly higher bridge strength in “anhedonia” than females (p = 0.044).

**Conclusions:**

This study revealed that insomnia, anxiety, and depression symptoms among youth are closely interconnected. Core symptoms such as “excessive worry” and “sleep dissatisfaction,” along with bridge symptoms like “fatigue,” “nervousness,” and “suicidal ideation,” represent potential intervention targets, with fatigue playing a dual role in the network. Males require particular attention regarding the intervention of “anhedonia.” Targeted improvement of these key symptoms may help break the cycle of comorbidity and provide precise directions for mental health interventions among young people.

## 1 Background

Depression, a global mental health issue, affects 350 million people worldwide and has become a significant cause of disability among adolescents and young adults (1). This common and serious mental health disorder is characterized by symptoms such as sad mood, anhedonia, lack of energy, and sleep disturbances (2). Adolescence is the phase of life stretching between childhood and adulthood, and its definition has long posed a conundrum. The ages of 10–24 years are a better fit with the development of adolescents nowadays (3). Young adulthood is a period marked by profound changes across physiological, psychological, social, and cognitive domains, and is recognized as a critical risk period for the onset of depression. Episodes occurring during this stage are often associated with high recurrence rates and relatively poorer functional outcomes (4). Global statistics show that 34% of adolescents are at risk of developing clinical depression (5). According to a meta - analysis of Chinese students, the prevalence of anxiety among college students is 26.0% (95% CI [19.0–34.0%]), and the prevalence of depression is 27.0% (95% CI [21.0–35.0%]) (6). Generalized anxiety disorder is characterized by feelings of threat, restlessness, irritability, sleep disturbance, and tension, and symptoms such as palpitations, dry mouth, and sweating (7). Anxiety disorders are the leading burden in mental health conditions for Chinese youth (8). Anxiety disorders not only significantly increase the suicide risk of youth (9), but are also closely associated with the occurrence of substance use disorders and depression (10). Youth depression and anxiety disorders have become major public health issues that urgently need to be addressed.

Sleep is a basic need, closely linked to youth development, particularly brain maturation (11). Insomnia is a common clinical condition characterized by difficulty initiating or maintaining sleep, accompanied by symptoms such as irritability or fatigue during wakefulness (12). Poor sleep is a risk factor for depressive symptoms (13). Research has indicated that 47.6% of patients at high risk for insomnia experience anxiety or depression (14), and 92% of patients with major depressive episode have significant sleep complaints (15). Additionally, previous studies have found that sleep disturbance exacerbates symptom severity in the majority of anxiety and related disorders (16). In China, a survey by Chen et al. indicated that 51.0% of youth sleep less than 8.0 hours per day on weekdays (17). Furthermore, sleep deprivation has been shown to be associated with depression, anxiety, poor academic performance, substance abuse, risky behaviors, and suicide among youth (18, 19). Insomnia, anxiety, and depression during youth may reflect shared underlying processes manifesting as different symptoms (20). Regarding insomnia, there is a significant gender difference among youth; a meta-analysis by Kocevska et al. revealed that the proportion of insomnia symptoms and sleep problems is significantly higher among female youth than male youth (21).

From an emotional perspective, insomnia is highly correlated with both anxiety and depressive disorders (22). Studies on insomnia disorder consistently show overlapping (salience network: insula and anterior cingulate cortex) and differential MRI correlation patterns between depressive symptoms (thalamus, orbitofrontal cortex, and their associated functional connectivity) and anxiety symptoms (functional connectivity associated with the default mode network). The insula has also been consistently identified as a brain region indicative of the severity of insomnia symptoms in depression (23). Another study conducted on young Japanese women found that individuals with depressive symptoms exhibited lower sleep efficiency and were more likely to experience daytime dysfunction (24). A large body of research both domestically and internationally has confirmed the close relationship between depressive and anxiety symptoms and sleep disturbances. These findings provide a foundation for considering the interconnections within the insomnia-anxiety-depression symptom network, suggesting that addressing sleep problems may serve as one approach to reducing emotional symptoms among youth.

Currently, most studies on the comorbidity of insomnia, depression, and anxiety are based on traditional latent variable theory, which interprets psychiatric symptoms as outcomes of underlying common factors. This approach typically uses total scores to represent the severity of depression and anxiety (25, 26). Unfortunately, such approaches may obscure meaningful associations between individual symptoms (27). In recent years, network analysis (NA), an emerging approach in psychopathology, has proven effective in visualizing and disentangling complex networks of psychological symptoms (28, 29). From a network perspective, symptoms are not interchangeable indicators but rather nodes within a causal system, with their roles determined by their position and connections within the network. Theorists suggest that highly “central” symptoms—those with stronger associations to other symptoms—are more likely to propagate activation throughout the network (30). Consequently, such symptoms are believed to play a key role in the onset and maintenance of mental disorders (30, 31). Several previous studies have used network analysis (NA) to explore the network structure of insomnia, anxiety, and depressive symptoms in various populations in China (e.g., high school students (32), nurses (33), residents of Macau (34), and adult patients with depression (35)), identifying core symptoms with more significant influence.

Currently, research on the relationship between insomnia and symptoms of depression and anxiety among youth remains insufficient. Addressing this gap, this study aims to systematically investigate the underlying patterns of association between insomnia metrics and symptoms of depression and anxiety in youth, as well as the role of gender, by applying network analysis (NA) to an integrated model of adolescent insomnia and anxiety-depression symptoms. This study seeks to answer the following key questions: How do insomnia and emotional symptoms such as anxiety and depression construct a dynamic associative network in youth? Which core nodes serve as “bridges” within this network? Do differences exist in network connectivity strength and the distribution of core nodes between male and female youth? By meticulously dissecting the mechanisms underlying the associations between insomnia and anxiety-depression through NA, this study aims to provide scientific evidence and practical references for early interventions targeting insomnia and emotional symptoms in youth.

## 2 Participants and methods

### 2.1 Participants

This study was conducted between October 2021 and December 2021, employing convenience sampling to select a total of 9,161 students from one university and one junior college in Jingzhou City, Hubei Province, and a junior college in Shangluo City, Shaanxi Province as participants. At the beginning of the project, we organized training and briefing sessions for the research personnel and teachers involved in the survey to ensure a unified and clear understanding of the investigation procedures. Subsequently, these researchers either relied on paid services or independently completed the distribution and collection of questionnaires. Throughout the survey process, researchers provided comprehensive guidance and answered questions. Questionnaires that were abandoned, took less than 10 minutes to complete, or had inconsistent semantics were excluded, resulting in a total of 7,247 valid questionnaires (valid response rate 79.11%). This study has been approved by the Ethics Committee of Jingzhou Mental Health Center (Ethics Approval Number: 2021LL0501).

### 2.2 Tools

#### 2.2.1 Self-compiled general information questionnaire

Adopt the Mental Health Status Survey Questionnaire for Adolescent Students compiled by Professor Maosheng Ran, which includes factors such as age, gender, grade level, major field of study, parents’ education level, family economic situation, academic pressure, and school life.

#### 2.2.2 Patient Health Questionnaire-9

The Chinese version of the PHQ-9 showed good reliability and validity in both the general population and youth (36, 37). This questionnaire evaluates depressive symptoms over the past two weeks, including anhedonia, sad mood, sleep problems, fatigue, appetite disturbance, guilt, difficulty concentrating, psychomotor problems, and thoughts of suicide or self-harm. Items on the PHQ-9 are scored on a Likert 4-point scale from 0 to 3 (0 = not at all, 1 = several days, 2 = more than half the days, 3 = nearly every day), with a total score of ≥5 indicating a positive assessment for depression, and higher scores indicating more severe depressive symptoms (38). In the present study, the Cronbach’s alpha coefficient value for the PHQ-9 was 0.92.

#### 2.2.3 Generalized Anxiety Disorder Scale-7

This study utilized the Chinese version of the GAD-7 scale, which has demonstrated high reliability and validity in the Chinese population (39–41). The scale is designed to comprehensively measure seven core anxiety symptoms, specifically including:: nervousness, uncontrollable worry, excessive worry, trouble relaxing, restlessness, irritability and feeling afraid. The GAD-7 scale follows a 4-point Likert scoring system, where 0 represents “not at all,” and 3 indicates “nearly every day,” with a total score of ≥5 indicating a positive assessment for anxiety, and higher scores indicating more severe anxiety symptoms. The Cronbach’s α for the GAD-7 in this study was 0.95.

#### 2.2.4 Insomnia Severity Index

The ISI is a self-report questionnaire consisting of 7 items aimed at assessing the severity of insomnia. The dimensions evaluated include: difficulty falling asleep, difficulty maintaining sleep, early morning awakening, satisfaction with current sleep patterns, impairment of daytime functioning due to sleep problems, the noticeability of sleep problems by others, and distress caused by sleep difficulties. Each item is rated on a 5-point Likert scale, with total scores ranging from 0 to 28. A total score of ≥8 indicates the presence of sleep problems, and higher scores indicate more severe insomnia (42). The Cronbach’s α coefficient for ISI in Chinese youth was 0.83, and the 2-week test–retest reliability was 0.79 (43). It has been validated among Chinese youth and demonstrates good psychometric properties (44, 45). The Cronbach’s α of this scale in this study was 0.90.

### 2.3 Methods

#### 2.3.1 Network estimation

Confirmatory factor analysis was employed to examine the factor structure of the PHQ-9 and ISI scales. Concurrently, Spearman correlation analysis was conducted to assess the strength of the association between PHQ-3 (sleep problems) and the total ISI score. A significant strong correlation was observed between PHQ-3 and the total ISI score (ρ = 0.74, p < 0.001). Consistent with previous studies (33, 46), this study identified substantial content overlap between PHQ-3 and insomnia symptoms assessed by the ISI; therefore, PHQ-3 was excluded from the subsequent analyses.

The network structure of insomnia, anxiety, and depression was estimated using the estimate network function in the R package bootnet (47). Due to ongoing controversies regarding the optimal approach for modeling scale item scores in network analysis (48), this study adopted a dichotomization method from prior research (49, 50), converting all items of the ISI, PHQ-9, and GAD-7 scales into binary values (‘0’ or ‘1’). Specifically, ‘0’ indicates the absence of insomnia, depressive, and anxiety symptoms, while ‘1’ signifies their presence. For all scales, non-zero values (i.e., any score greater than 0) were recoded as ‘1’, whereas a score of ‘0’ retained its original meaning. An Ising model was used to assess the depressive-anxiety symptoms network structure based on binary data (49, 51). Briefly, an Ising model can be conceived as a series of pairwise associations between binary variables, after controlling for the confounding effects of all other associations.

To identify the most central (influential) symptoms within a network model of insomnia, anxiety, and depression, we estimated strength centrality. Strength centrality, defined as the sum of absolute edge weights connected to a node, indicates how strongly a node is associated with others (31). For detecting symptoms that link two or more distinct disorders, bridge strength metrics were utilized. Bridge strength for each node was calculated using the bridge function from the networktools R package (52). A cutoff threshold of 80% of the maximum observed bridge strength value in the dataset was applied to identify bridge symptoms (53). In addition, the predictability of each node was estimated using the R package mgm. Predictability refers to the extent to which the variation in a given node can be explained by the variation in its neighboring nodes within the network. The average predictability across all nodes reflects the degree to which the network is influenced by external factors. A higher average predictability indicates that the network structure is largely self-explanatory, with less variance being attributable to external influences. Predictability is visually represented in the network plot by the size of the circular area around each node.

#### 2.3.2 Network accuracy and stability

To assess the accuracy and stability of the observed network model, R package bootnet (version 1.6) was used based on 1000 bootstraps performed for each node. Based on nonparametric bootstrapping, a new dataset with 95% probability confidential intervals (CIs) was generated to assess the accuracy of edge weights (31). Results with low overlaps of CIs indicate more accurate edge weights. Stability strength was also evaluated using the correlation stability(CS) coefficient. A CS coefficient greater than 0.25 indicates acceptable stability of node centrality, while a coefficient greater than 0.5 suggests good stability (47). Finally, differences of each pair of edges or nodes were estimated using a nonparametric bootstrapped method based on CIs with 95% probabilities. Statistically significant differences between each pair of edges or nodes were suggested by the inclusion of zero in the CIs.

#### 2.3.3 Network comparison

To examine the moderating effects of gender, COVID-19 quarantine status, and family income on insomnia, anxiety, and depressive symptoms among young people, we compared the insomnia-depression-anxiety network models across different subgroups defined by gender (male vs. female), quarantine status (quarantined vs. non-quarantined), and family income (better vs. poorer). For these analyses, we utilized the R package “NetworkComparisonTest” (version 2.2.2) (54) to conduct 1,000 permutations, which is a permutation test designed to assess differences between two networks. The Network Comparison Test (NCT) was performed on the subgroups (i.e., male vs. female, quarantined vs. non-quarantined, better family income vs. worse family income) with 1,000 permutations to evaluate the global network strength (the sum of the absolute values of all edge weights) and network structure (the distribution of edge weights). Additionally, multiple comparisons were conducted using Bonferroni corrections to assess the strength of each edge between the two networks.

## 3 Results

### 3.1 Descriptive statistics

In this survey, there were 2970 (40.98%) males and 4277 (59.02%) females, with an average age of 20.25 ± 1.21 years. The participants included 2406 (33.20%) freshmen, 3861 (53.28%) sophomores, 770 (10.63%) juniors, and 210 (2.90%) seniors. The positive rates of ISI, GAD-7, and PHQ-9 were 28.86%, 25.12%, and 32.73% among males, compared with 28.10%, 26.61%, and 31.66% among females, respectively.

The results comparing the factor scores of the ISI, GAD-7, and PHQ-9 scales and gender differences are shown in **Table 1**. Females scored higher than males on the ISI1 and ISI4 factors, with scores of 0.57 ± 0.84 and 1.40 ± 0.99 compared to 0.56 ± 0.91 and 1.33 ± 1.05 (P < 0.01), respectively, while there were no significant gender differences in other ISI factors (P > 0.05). In the PHQ-9 scale, females scored higher than males on PHQ2, PHQ4, and PHQ5 (P < 0.05), while scoring lower on PHQ1, PHQ8, and PHQ9 factors (P < 0.01). In the comparison of factor scores on the GAD-7 scale, females scored higher than males on the GAD1, GAD2, GAD3, GAD4, GAD6, and GAD7 factors (P < 0.05).

**Table 1:** Gender differences in scale scores.

Item abbreviation	Male N = 2,970^1^	Female N = 4,277^1^	p-value^2^
ISI1	0.56 (0.91)	0.57 (0.84)	**0.006**
ISI2	0.41 (0.81)	0.40 (0.75)	0.352
ISI3	0.48 (0.84)	0.49 (0.83)	0.171
ISI4	1.33 (1.05)	1.40 (0.99)	**0.002**
ISI5	0.96 (0.99)	0.96 (0.94)	0.460
ISI6	0.86 (0.97)	0.81 (0.92)	0.074
ISI7	0.76 (0.96)	0.73 (0.94)	0.289
PHQ1	0.45 (0.71)	0.42 (0.67)	**0.047**
PHQ2	0.47 (0.70)	0.49 (0.68)	**0.006**
PHQ3	0.46 (0.75)	0.49 (0.74)	**0.017**
PHQ4	0.53 (0.75)	0.55 (0.73)	**0.036**
PHQ5	0.45 (0.75)	0.50 (0.77)	**0.002**
PHQ6	0.45 (0.72)	0.45 (0.71)	0.755
PHQ7	0.39 (0.69)	0.39 (0.68)	0.847
PHQ8	0.31 (0.63)	0.24 (0.57)	**<0.001**
PHQ9	0.19 (0.50)	0.16 (0.48)	**<0.001**
GAD1	0.40 (0.65)	0.49 (0.66)	**<0.001**
GAD2	0.33 (0.63)	0.38 (0.65)	**<0.001**
GAD3	0.43 (0.70)	0.50 (0.69)	**<0.001**
GAD4	0.37 (0.67)	0.40 (0.66)	**0.010**
GAD5	0.29 (0.60)	0.27 (0.56)	0.142
GAD6	0.35 (0.64)	0.40 (0.67)	**<0.001**
GAD7	0.34 (0.64)	0.36 (0.63)	**0.017**

^1^Mean (SD).

^2^Wilcoxon rank sum test.

ISI, Insomnia Severity Index; PHQ-9, Patient Health Questionnaire-9; GAD-7, Generalized Anxiety Disorder Scale-7.

Bold indicates p<0.05.

### 3.2 Network structure and node centrality analysis of insomnia, anxiety, and depression symptoms

**Figure 1:**
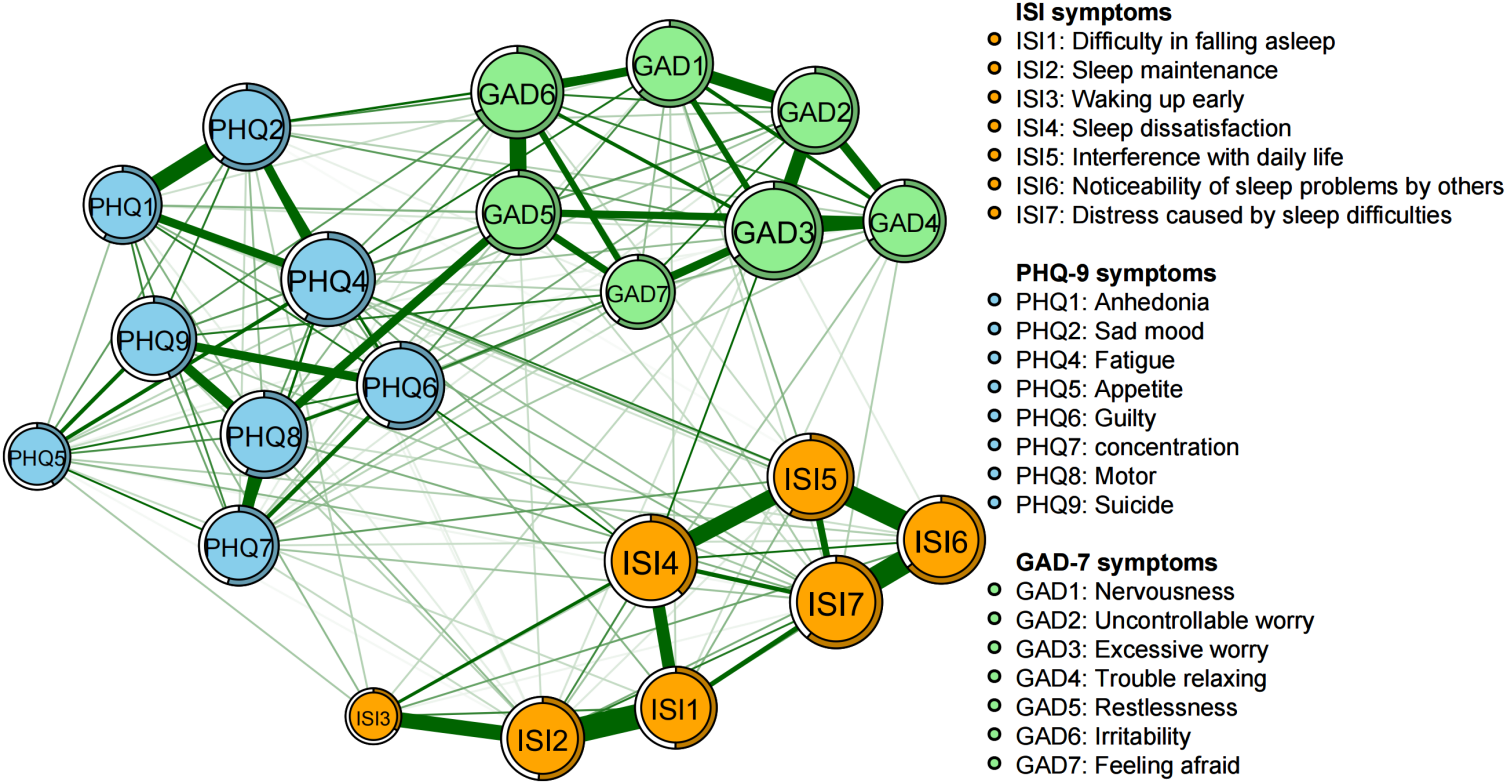
Network structure of insomnia, anxiety, and depressive symptoms among youth. In the diagram symptom node with stronger connections are closer to each other. The orange node denotes the ISI items; the skyblue node denotes the PHQ-9 items; the light green node denotes the GAD-7 items. The dark green lines represent positive correlations. The edge thickness represents the strength of the association between symptom nodes.

**Figure 1** displays the network structure of insomnia, anxiety, and depressive symptoms among all participants. From the network structure, it can be observed that the nodes representing insomnia, anxiety, and depression cluster separately, forming three distinct communities. The predictability of each symptom is visualized using circular pie charts. The node predictability values range from 33.6% to 68.4%, with an average of 56.3%, indicating that on average, 56.3% of the variance in each node within the network can be explained by its adjacent nodes (**Figure 1**). Although approximately half of the variance in each symptom can be explained by other nodes in the network, this also implies that the remaining variance cannot be fully accounted for by mutual interactions between symptoms.

**Figure 2:**
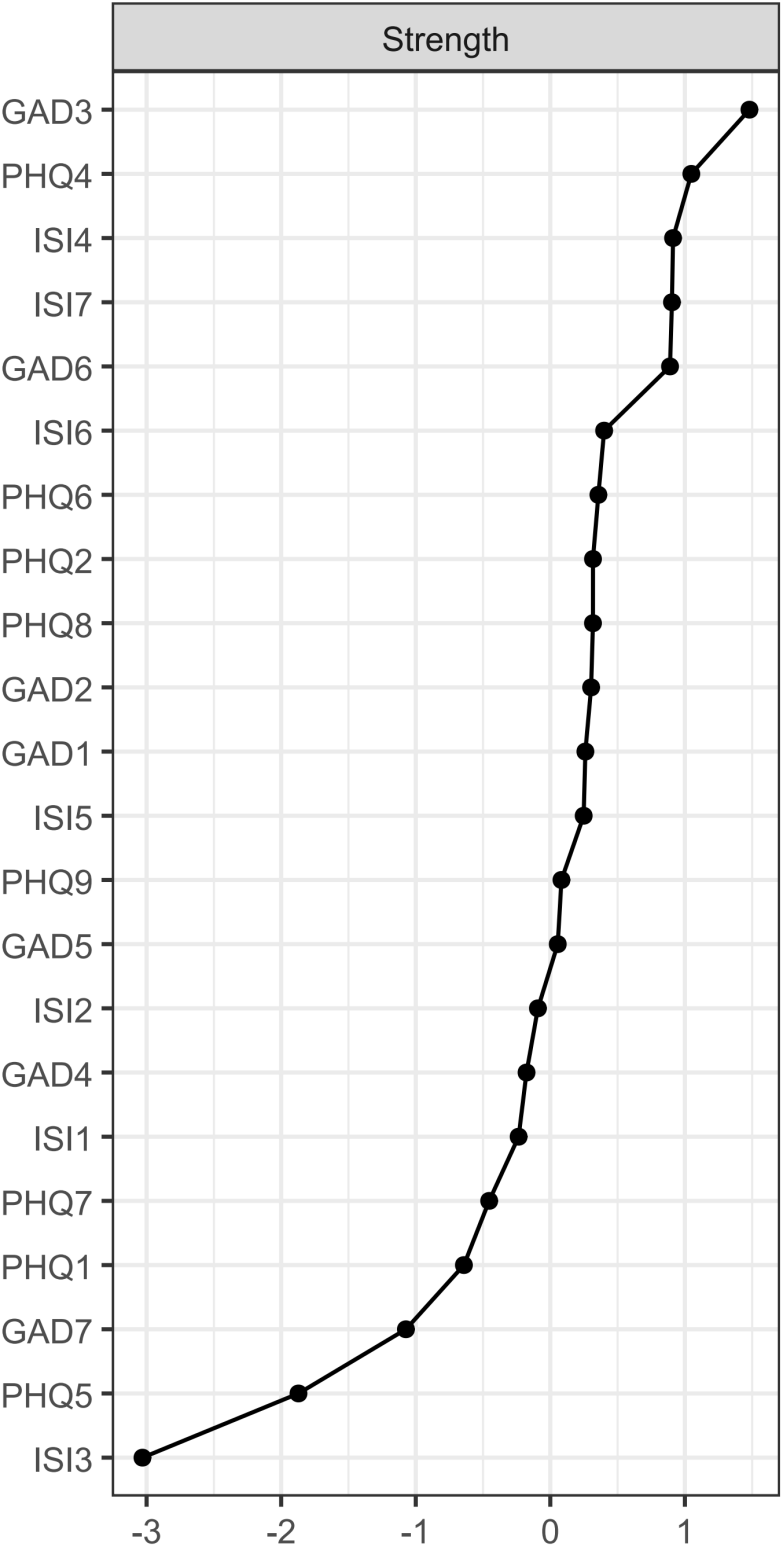
Node strength in the insomnia, anxiety, and depression network.

**Figure 3:**
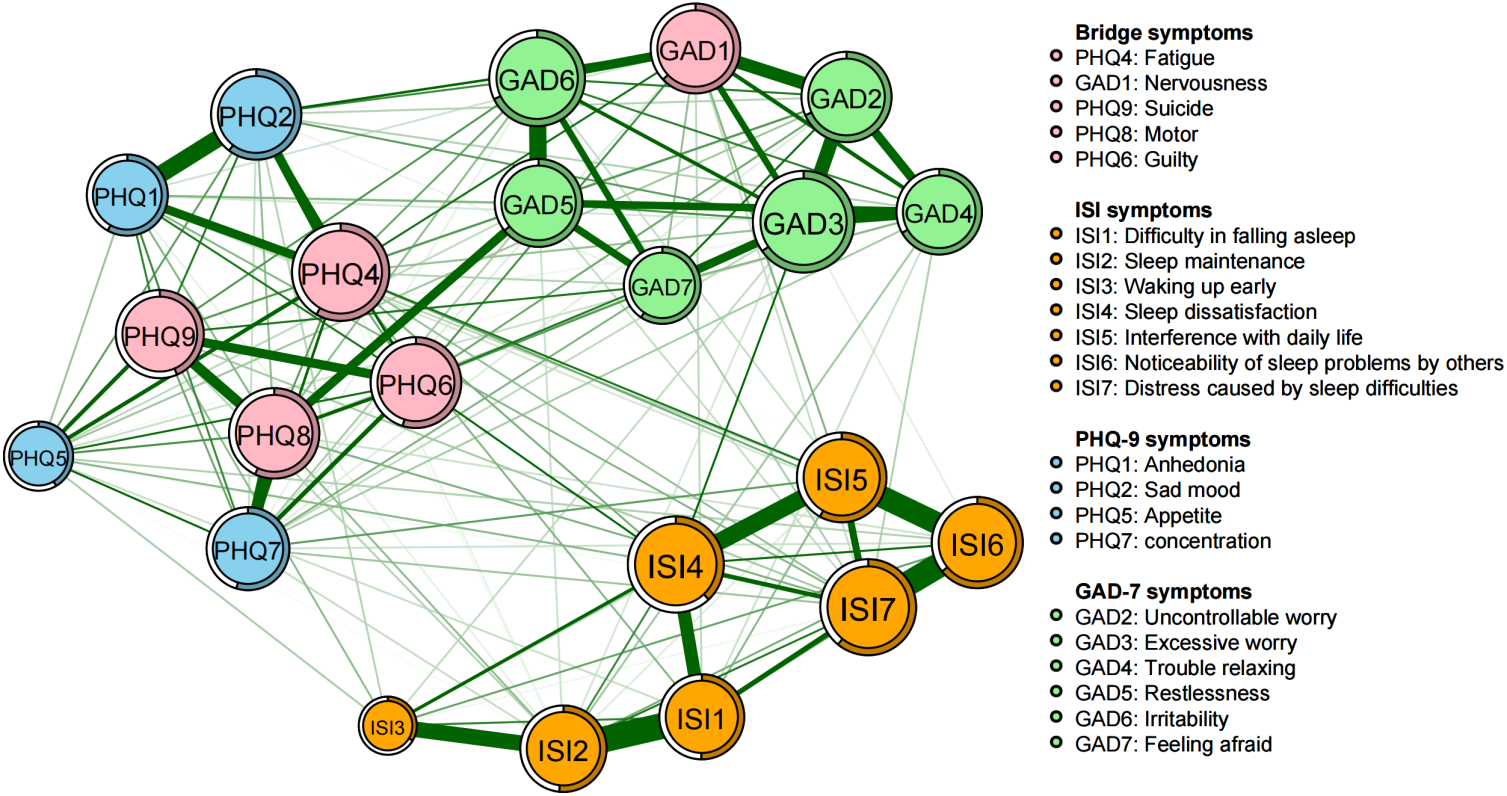
Network structure of insomnia, anxiety, and depressive symptoms showing bridge symptoms in youth.

**Figure 4:**
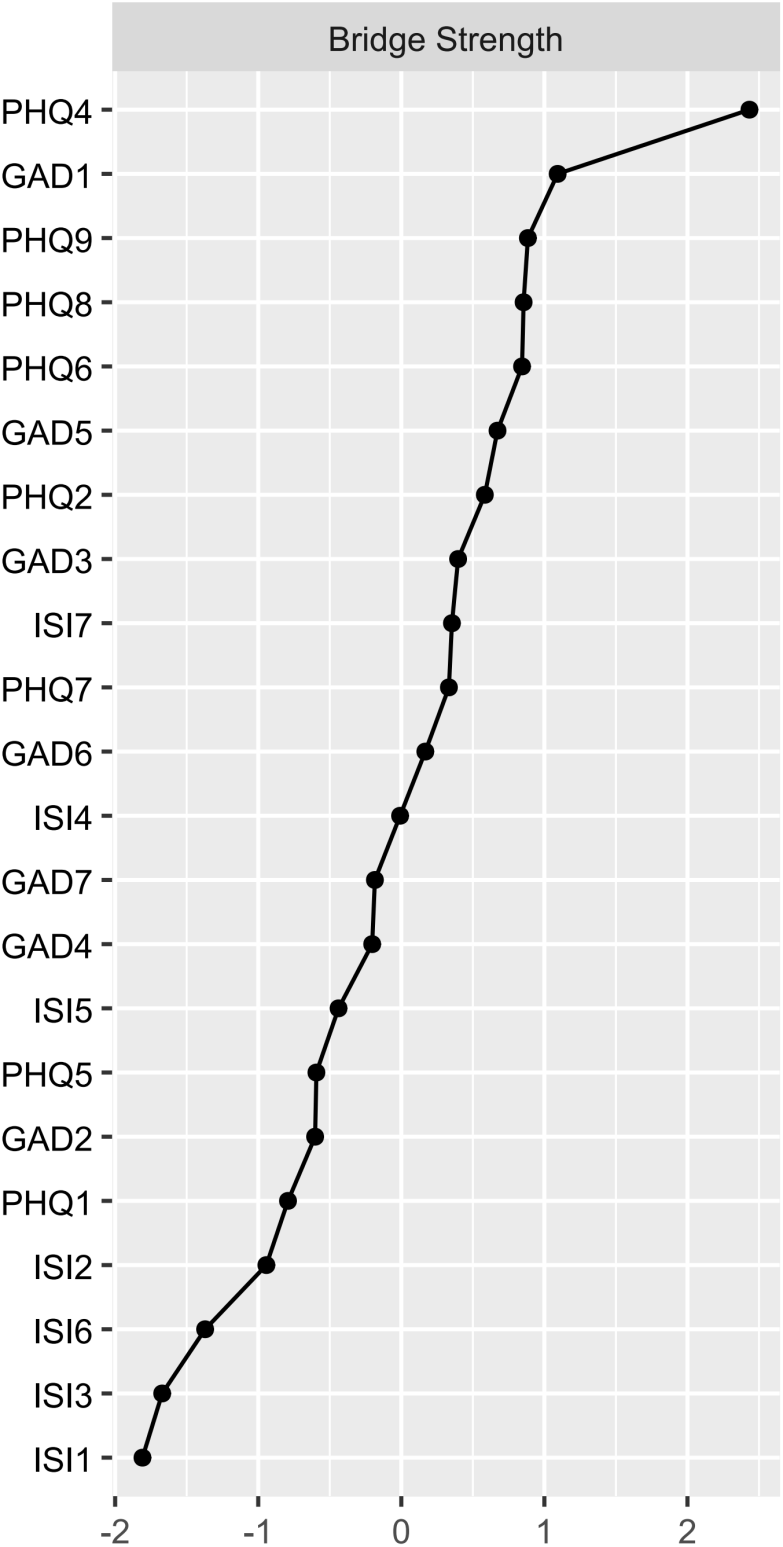
Bridge strength in the insomnia, anxiety, and depression network.

The symptoms of “excessive worry” (GAD3), “fatigue” (PHQ4), “sleep dissatisfaction” (ISI4), and “distress caused by sleep difficulties” (ISI7) are centrally positioned in terms of node strength, demonstrating strong connectivity (**Figure 2**). The intensity of bridge symptoms identified as bridges in this sample, ranked from highest to lowest, are: “fatigue” (PHQ4), “nervousness” (GAD1), “suicidal ideation” (PHQ9), “psychomotor problems” (PHQ8), and “guilt” (PHQ6) (**Figures 3**, **4**).

The edge correlation between ISI1 “Difficulty in falling asleep” and ISI2 “Sleep Maintenance” is the strongest, followed by the pairs ISI6-ISI7 (Noticeability of sleep problems by others - Distress caused by sleep difficulties), ISI5-ISI6 (Interference with daily life - Noticeability of sleep problems by others), ISI4-ISI5 (Sleep dissatisfaction - Interference with daily life), GAD5-GAD6 (Restlessness - Irritability), and PHQ1-PHQ2 (Anhedonia - Sad mood) (**Figure 1**, **Supplementary Figure S1**).

### 3.3 Network stability and accuracy

**Figure 5:**
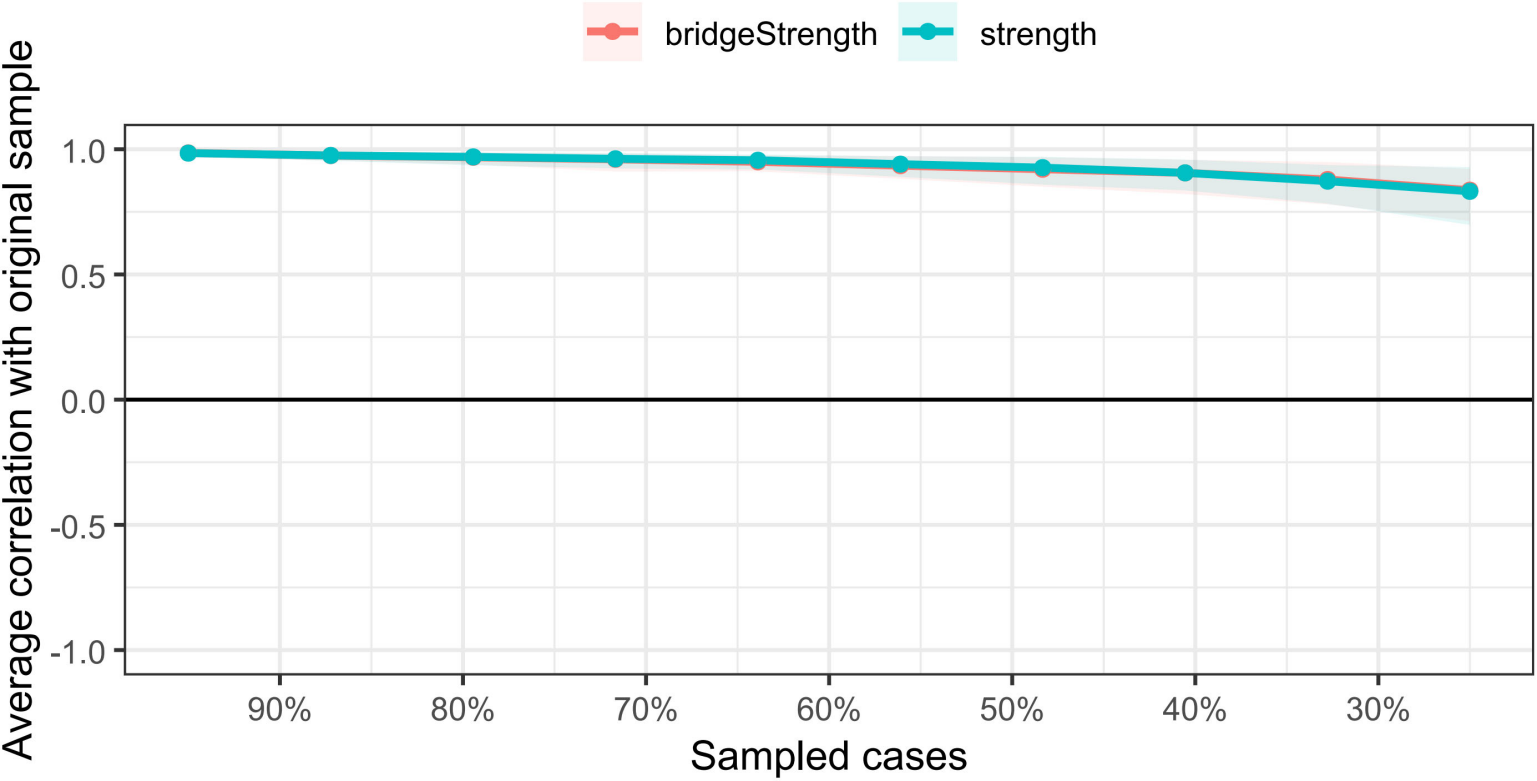
The stability of centrality and bridge centrality indices using case-dropping bootstrap. The x-axis represents the percentage of cases of the original sample used at each step. The y-axis represents the average of correlations between the centrality indices in the original network and the centrality indices from the re-estimated networks after excluding increasing percentages of cases. The line indicates the correlations of strength and bridge strength.

The correlation stability coefficients for both node strength and bridge strength exceeded 0.75, indicating good network stability (**Figure 5**). The results of the nonparametric bootstrap procedure revealed that most comparisons between node strength and bridge strength were statistically significant (**Supplementary Figures S2**, **S3**). Additionally, the bootstrapped 95% confidence intervals (CIs) were narrow, indicating trustworthy edge results (**Supplementary Figure S1**).

### 3.4 Results of network comparison

**Figure 6:**
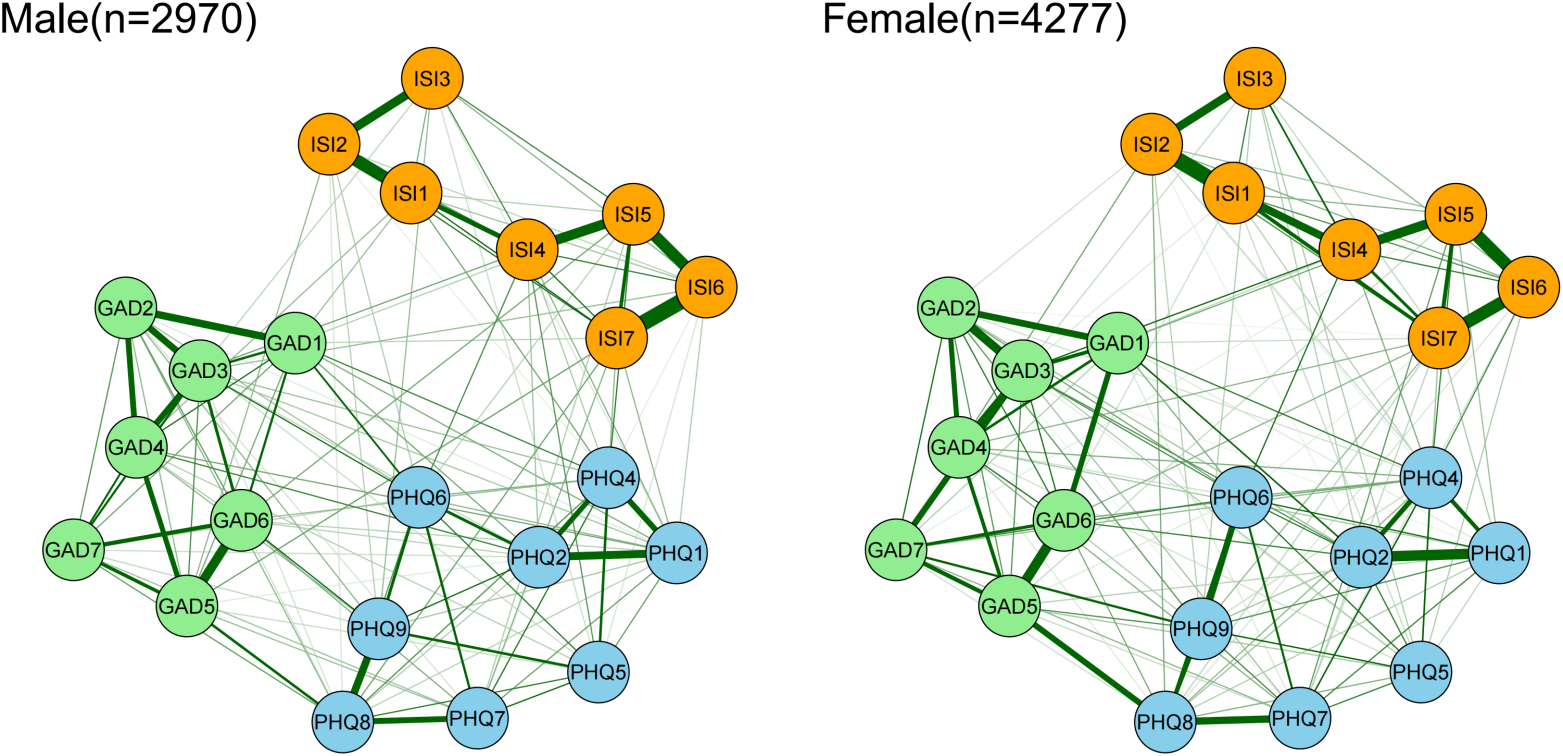
Network comparison of insomnia, anxiety, depressive symptoms between genders.

NCT was conducted between different genders. The network structures of insomnia and depression, anxiety symptoms in male and female populations are shown in **Figure 6**. The results indicate significant gender differences in both network structural invariance (M = 0.909, p = 0.025) and global strength (Female: 70.527, Male: 75.155; S = 4.628, p = 0.041) as depicted in **Supplementary Figure S4**. Males exhibited significantly higher bridge strength in “anhedonia” (PHQ1) compared to females (P = 0.044). These results suggest that the synergistic effects among symptoms are stronger in males, with more pronounced individual bridge strength centrality.

A comparison of the networks generated based on whether individuals were quarantined due to the COVID-19 pandemic did not reveal significant differences in global network strength (Quarantined: 59.881 vs Non-quarantined: 73.509; S = 13.628, p = 0.234), network structural invariance (M = 1.168, p = 0.077), or individual edge weights (all p-values > 0.05 after Bonferroni correction). Similarly, no significant differences were found in global network strength (Better income: 73.60 vs Poorer income: 66.71; S = 6.89, p = 0.335), network structural invariance (M = 0.92, p = 0.063), or individual edge weights (all p-values > 0.05 after Bonferroni correction) based on household income. Relevant figures are presented in the **Supplementary Materials** (**Supplementary Figures S5**, **S6**).

## 4 Discussion

This study employed network analysis to investigate the relationships among insomnia, depression, and anxiety in young students. All results demonstrated stability and accuracy, indicating strong correlations among the internal symptoms of these three mental health disorders.

In this study, “excessive worry” (GAD3), “fatigue” (PHQ4), “sleep dissatisfaction” (ISI4), and “distress caused by sleep difficulties” (ISI7) occupied central positions in terms of node strength, potentially serving as key nodes linking insomnia with anxiety and depressive disorders. To improve sleep, particular attention should be paid to “sleep dissatisfaction” and “distress caused by sleep difficulties,” as these two symptoms reflect individuals’ subjective experiences regarding sleep issues. Research has confirmed that dysfunctional beliefs are a core mechanism in the maintenance of insomnia, emphasizing the importance of targeted changes to these beliefs in treatment (55). Regular physical exercise may also help improve sleep quality (56), contributing to better satisfaction with sleep. Additionally, it has been found that mindfulness meditation can alleviate distress caused by sleep issues. Awareness and acceptance may be mechanisms through which mindfulness interventions improve sleep quality, partly by reducing psychological stress (57).

“Excessive worry” (GAD3) being identified as a core symptom within the insomnia, anxiety, and depression symptom network aligns with previous research findings (58). As the cognitive core of anxiety, “excessive worry” is associated with anxious and depressive moods as well as sleep disturbances (59). There exists a bidirectional relationship between worry and sleep quality; worry can affect sleep quality, which in turn can exacerbate worry (60). In a study conducted in South Korea, participants who experienced poor sleep quality exhibited higher levels of anxiety regardless of the duration of their sleep (61). A randomized controlled trial found that preventive interventions targeting worry and rumination among adolescents and youth could reduce the levels and prevalence of anxiety and depressive symptoms (62).

We found that “fatigue”(PHQ4) not only serves as a core symptom within the network structure of our sample but is also the most prominent bridge symptom. This finding aligns with previous research on Filipino migrant domestic workers (53), suggesting that it may be a common critical factor in mental health issues across different populations, warranting focused attention and intervention. “Fatigue” is characterized by prolonged loss of energy, exhaustion, or feelings of weakness (63), and is a shared feature of both depression and insomnia (64). A cohort study involving over 4,000 American adolescents found that sleep deprivation increased the risk of depression by 25% to 38% (65). An Australian clinical trial indicated a high correlation between sleep disorders and depressive states, and associated poor treatment response among adolescents with depression (66). Lovato et al.’s research discovered that depressed youth experience longer sleep onset latency, more awakenings after sleep onset, and lower sleep efficiency (67). Individuals experiencing fatigue typically report more severe insomnia symptoms and depression compared to those without fatigue (68). Given that depression acts as a significant mediator between insomnia and fatigue, controlling for either insomnia or depression might alleviate fatigue in individuals with insomnia. However, attempts to arbitrarily extend sleep duration could exacerbate their fatigue (68).

“Nervousness” (GAD1) exhibited high bridge strength within the network of insomnia, anxiety, and depression symptoms among young individuals. This finding suggests that tension is more susceptible to changes in other symptoms, and/or more likely to influence other symptoms in return. As a core symptom of anxiety, tension involves complexity across emotional, physiological, and cognitive dimensions. Due to frequent exposure to negative events and feelings of insecurity, tension and anxiety are prevalent among youth (69, 70). This result is consistent with recent studies conducted in China (71), which found that “nervousness” served as a bridging symptom between depression and anxiety among nursing students during the COVID-19 pandemic.

“Suicidal ideation” (PHQ9), “psychomotor problems” (PHQ8), and “guilt” (PHQ6) are other bridge symptoms within the network of our sample. Previous studies have found that adolescents with higher levels of insomnia report a greater frequency of non-suicidal self-injury behaviors (72). This finding aligns with Tao et al.’s research, which used suicidal ideation as a grouping criterion and indicated that the most influential symptom directly associated with “suicidal ideation” is “guilt” (41). Prior studies by Cai et al. and Tao et al. also discovered that “Suicidal ideation” (PHQ9) and “guilt” (PHQ6) serve as bridging symptoms in the connection between depression and anxiety among youth (50, 73). In contrast, Kaiser et al.’s study on adult inpatient samples demonstrated that psychomotor agitation or retardation serves as the strongest bridge node between anxiety and depression (74). These differences across studies may partly be attributed to variations in sample populations. Given the significant differences in sociodemographic characteristics and related life stressors among these study samples, the aforementioned symptoms appear to be hallmark bridge symptoms contributing to the comorbidity of insomnia, anxiety, and depression. Therefore, interventions targeting these key symptoms hold important clinical significance (75).

The pattern of symptom associations among insomnia, depression, and anxiety exhibits fundamental differences between males and females. Overall, the inter-symptom connectivity is stronger in males, suggesting that males may form a more tightly connected “symptom cluster,” in which changes in one symptom are more likely to trigger a cascade of other symptoms. Notably, “anhedonia” (loss of interest or pleasure in activities) functions as a stronger cross-domain bridge symptom in the male network, indicating that it may serve as a key hub linking insomnia with anxiety and depression. For instance, insomnia may lead to anhedonia, which in turn exacerbates depressive and anxious symptoms, forming a vicious cycle. Tao et al. found that anhedonia was most prevalent among college students (76). This gender difference may be influenced by multiple factors, including biological, sociocultural, and psychological mechanisms.

Testosterone distribution and secretion differ markedly between males and females, affecting the neurotransmitter systems through various pathways, including dopamine, serotonin, and gamma-aminobutyric acid (GABA) systems. For instance, testosterone enhances dopamine release in the mesolimbic system, counteracting anhedonia caused by depression (77). Female gonadal hormones, particularly estrogen and progesterone, play a crucial role in regulating mood, cognition, and overall brain health (78). Sex hormones significantly influence the onset and maintenance of anxiety, impacting biological, behavioral, and cognitive processes. High levels of estradiol and progesterone can be either protective or increase vulnerability depending on cognitive or behavioral processes during hormonal changes (79). There are significant differences in emotional expression among college students influenced by factors such as gender, cultural background, and personality traits (80). Males tend to express psychological distress more through physical symptoms (81) and exhibit less help-seeking behavior for social support (82). The association and impact of depressive mood with feelings of self-blame and somatic symptoms are more pronounced in males than in females (83). Emotional disorders and anxiety in males are also more susceptible to parenting styles (84). Behavioral patterns in response to stress also differ significantly between men and women. Men are more likely to use avoidance coping strategies, while women are more prone to rumination, tending to “internalize” emotions through repetitive thinking or self-critical behaviors (85, 86). This aligns with Nolen-Hoeksema’s theory of gender differences in rumination (87, 88). These gender differences may relate to specificities in stress coping and emotion regulation in males, potentially leading to more pronounced interactions between depressive and anxious symptoms, as well as more prominent cumulative effects of sleep problems. This study, along with Tao et al.’s research, has also identified significant differences in network structures between males and females. They further found that males exhibited significantly higher strength in symptoms of difficulty concentrating and sleep deprivation compared to females (89). However, these findings contrast with a recent study examining the network characteristics of comorbid anxiety and depression among Chinese first-year university students (n=2082), which did not observe significant gender differences in network structure (71). This discrepancy may be attributed to differences in sample populations.

Tailoring mindfulness training strategies based on gender differences and the unique personal and physiological characteristics of young men and women can help enhance psychological resilience, thereby providing more effective prevention and intervention for youth emotional issues. For male populations, in addition to conventional treatments, behavioral activation combined with group exercise interventions could be considered. Exercise-induced endogenous testosterone can help alleviate anhedonia, and physical activity is also a highly effective factor in enhancing stress resistance (90). It is recommended to encourage young students to engage in regular physical exercise, which not only directly reduces anxiety and other emotional issues, improving physical and mental health but also indirectly promotes mental health by fostering resilience (91, 92). Additionally, developing more suitable active mindfulness training methods, such as mindful yoga or Tai Chi, could better cater to their externalizing coping styles (93).

The clinical manifestations of insomnia are often comorbid with mental health disorders, particularly anxiety and depression. However, more than 40% of practicing physicians “at least somewhat agree” that the treatment of comorbid insomnia should focus solely on the underlying mental health condition, indicating a significant gap between current clinical practices and evidence-based guidelines (94). A review examining the effects of pharmacotherapy for fatigue in major depressive disorder found that medications with dopaminergic and/or noradrenergic actions were most effective in improving symptoms of fatigue and energy deficiency (64). Beyond pharmacotherapy, psychotherapy has been shown to significantly improve sleep quality among young university students (95). A previous study using dynamic network intervention analysis explored the specific symptom improvement sequence triggered by Cognitive Behavioral Therapy for Insomnia (CBT-I) during treatment. It was found that CBT-I interventions typically address sleep behavioral issues (such as difficulty falling asleep and sleep maintenance problems) in the initial stages, followed by improvements in subjective sleep dissatisfaction (such as dissatisfaction with sleep quality) after four weeks of treatment. The researchers concluded that CBT-I improves both insomnia and depressive symptoms primarily by directly addressing core sleep problems, then indirectly influencing other symptoms through their interconnections within the symptom network (96). In the 2023 edition of the European Guidelines for Insomnia, CBT-I is recommended as the first-line treatment for chronic insomnia in adults of any age, including those with comorbid conditions, with pharmacotherapy considered only if CBT-I proves ineffective (97). The efficacy of CBT-I has been extensively studied, showing short-term effectiveness (within two weeks) comparable to sleeping pills and superior long-term effectiveness (over one year) compared to medication, with minimal side effects and virtually no serious adverse reactions (98). Furthermore, a systematic review and meta-analysis by Li et al. indicated that adding 4–12 weeks of mindfulness therapy to standard treatments can significantly improve symptoms of anxiety and depression across different age groups suffering from insomnia, anxiety, and depression (99), although it faces limitations due to high human resource costs and resource consumption. Recent preliminary studies have confirmed that virtual reality (VR) therapy can significantly improve sleep quality, reduce symptoms of depression and anxiety, and enhance cognitive function and autonomic regulation in patients with chronic insomnia (100). Integrating VR technology into CBT may address these limitations, making it a promising approach worthy of promotion in routine clinical practice (101).

Despite these potential applications, several limitations of the current study must be acknowledged: Firstly, the study employed a cross-sectional design, which cannot examine the dynamic and temporal relationships among symptoms over time. Secondly, the data were collected from a specific cohort of Chinese young students; different time periods and/or student populations may exhibit different network structures. Thirdly, the study utilized convenience sampling, which limits the generalizability of the findings to the broader population of young students. Fourthly, reliance on self-report instruments may introduce potential biases, including inaccuracies in recall and response biases. These limitations underscore the need for future research to adopt longitudinal designs and multi-modal assessment methods. Such improvements would enable a more comprehensive understanding of the complex interactions between youth sleep problems and symptoms of anxiety and depression, providing a stronger foundation for developing effective interventions.

Through network analysis, this study found that “excessive worry,” “fatigue,” “sleep dissatisfaction,” and “distress caused by sleep difficulties” are core nodes within the symptom network of insomnia, anxiety, and depression among young students. The high node strength of these symptoms suggests that they play a key role in the development and maintenance of transdiagnostic comorbidity and should be prioritized as intervention targets. Multilevel interventions targeting “bridge symptoms” (e.g., fatigue, nervousness, suicidal ideation, psychomotor problems, and guilt), such as cognitive behavioral therapy combined with mindfulness training and physical activity interventions, may effectively reduce the comorbid burden of insomnia and emotional disorders by disrupting the chain reactions between symptoms. These findings offer practical implications for building a mental health protection system for youth: focusing on the early identification and targeted intervention of core symptoms can significantly reduce the risk of sleep problems transforming into anxiety and depression, thereby holding important public health significance for improving the mental health of young populations.

## Data Availability

The raw data supporting the conclusions of this article will be made available by the authors, without undue reservation.

## Supplementary material

Supplementary Figure 1 Bootstrapped confidence intervals of edge weights.

Supplementary Figure 2 Bootstrapped stability test for ‘node strength’. Bootstrapped difference tests between node strength of insomnia, anxiety, and depressive symptoms. Gray boxes indicate nodes that do not significantly differ from one another. Black boxes indicate nodes that differ significantly from one another (α = 0.05). White boxes show the values of node strength.

Supplementary Figure 3 Bootstrapped stability test for ‘bridge strength’. Bootstrapped difference tests between bridge strength of insomnia, anxiety, and depressive symptoms. Gray boxes indicate nodes that do not significantly differ from one another. Black boxes indicate nodes that differ significantly from one another (α = 0.05). White boxes show the values of bridge strength.

Supplementary Figure 4 Comparison of network properties between females and males. There was a significant difference in network global strength between females and males (Female: 70.527 vs. Male: 75.155; S = 4.628, p = 0.041), as well as in network structural invariance (M = 0.909, p = 0.025).

Supplementary Figure 5 Comparison of global strength and edge weights between quarantined and non-quarantined individuals.

Supplementary Figure 6 Comparison of global strength and edge weights between groups with better and poorer family income.
