# Supplementary figures and images for: Network characteristics of the youth’s insomnia and emotional symptoms and their gender differences
Source: Front Psychiatry. 2025 Jun 16;16:1597652. doi: 10.3389/fpsyt.2025.1597652 (PMC12206766; doi:10.3389/fpsyt.2025.1597652)

● Bootstrap mean ● Sample

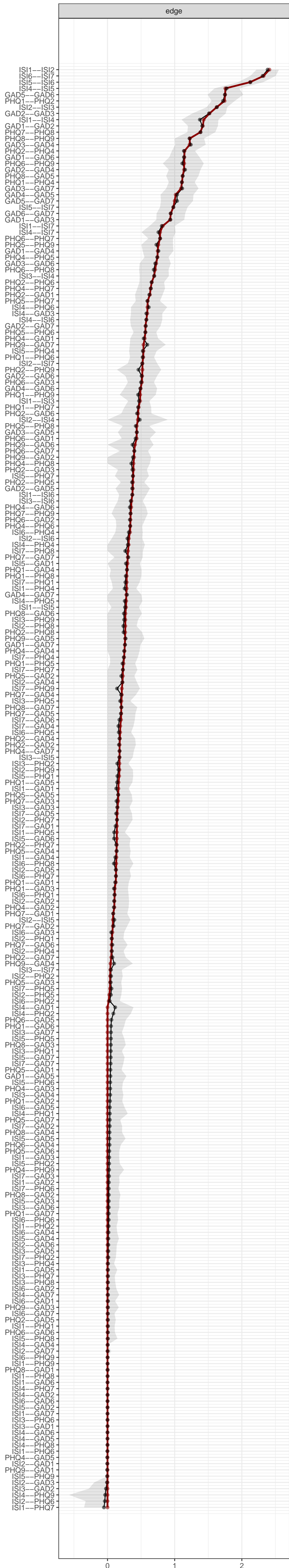

Supplement: Supplementary Figure 1 — Bootstrapped confidence intervals of edge weights. [file Image1.pdf]

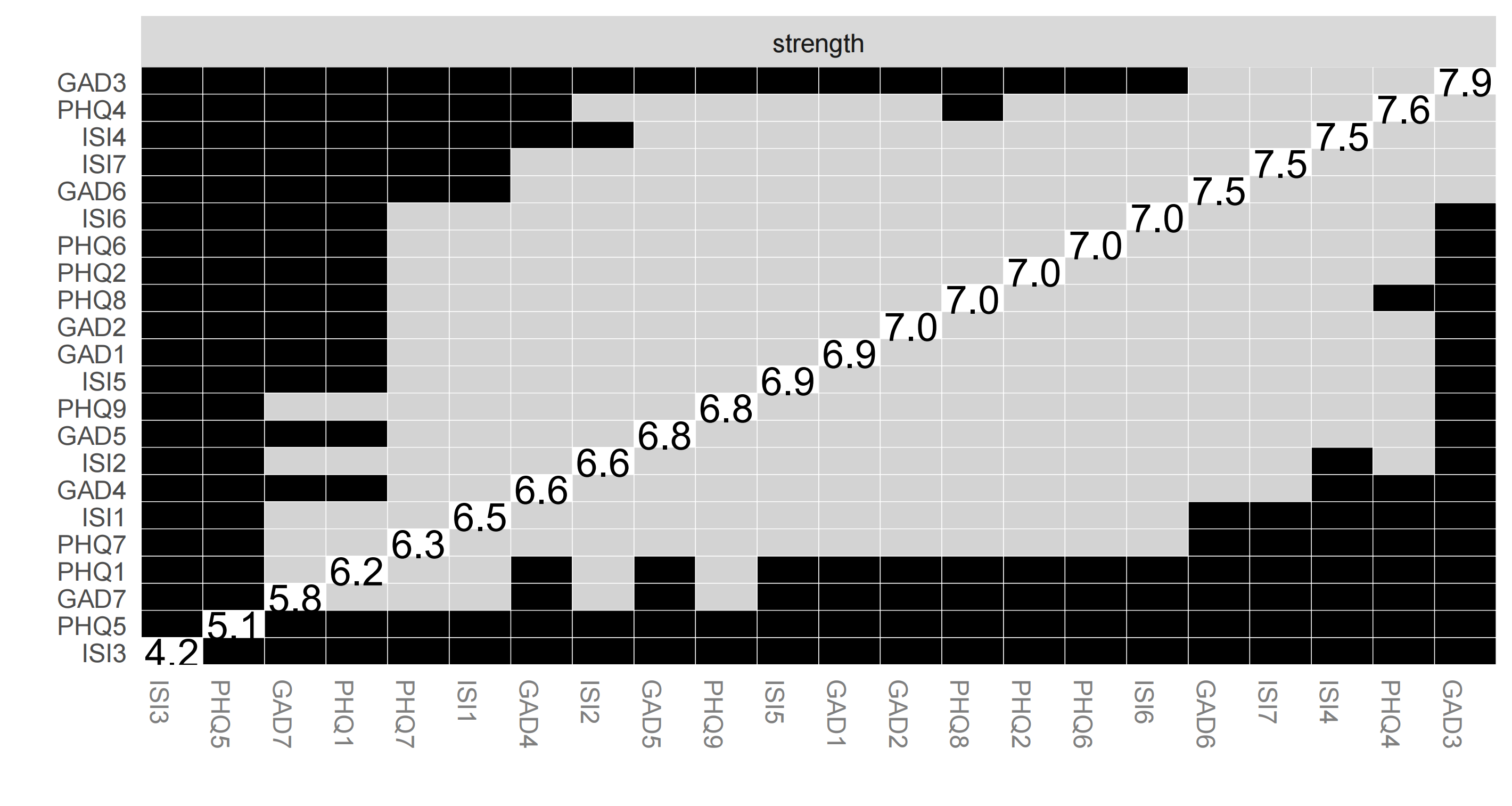

Supplement: Supplementary Figure 2 — Bootstrapped stability test for ‘node strength’. Bootstrapped difference tests between node strength of insomnia, anxiety, and depressive symptoms. Gray boxes indicate nodes that do not significantly differ from one another. Black boxes indicate nodes that differ significantly from one another (α = 0.05). White boxes show the values of node strength. [file Image2.tif]

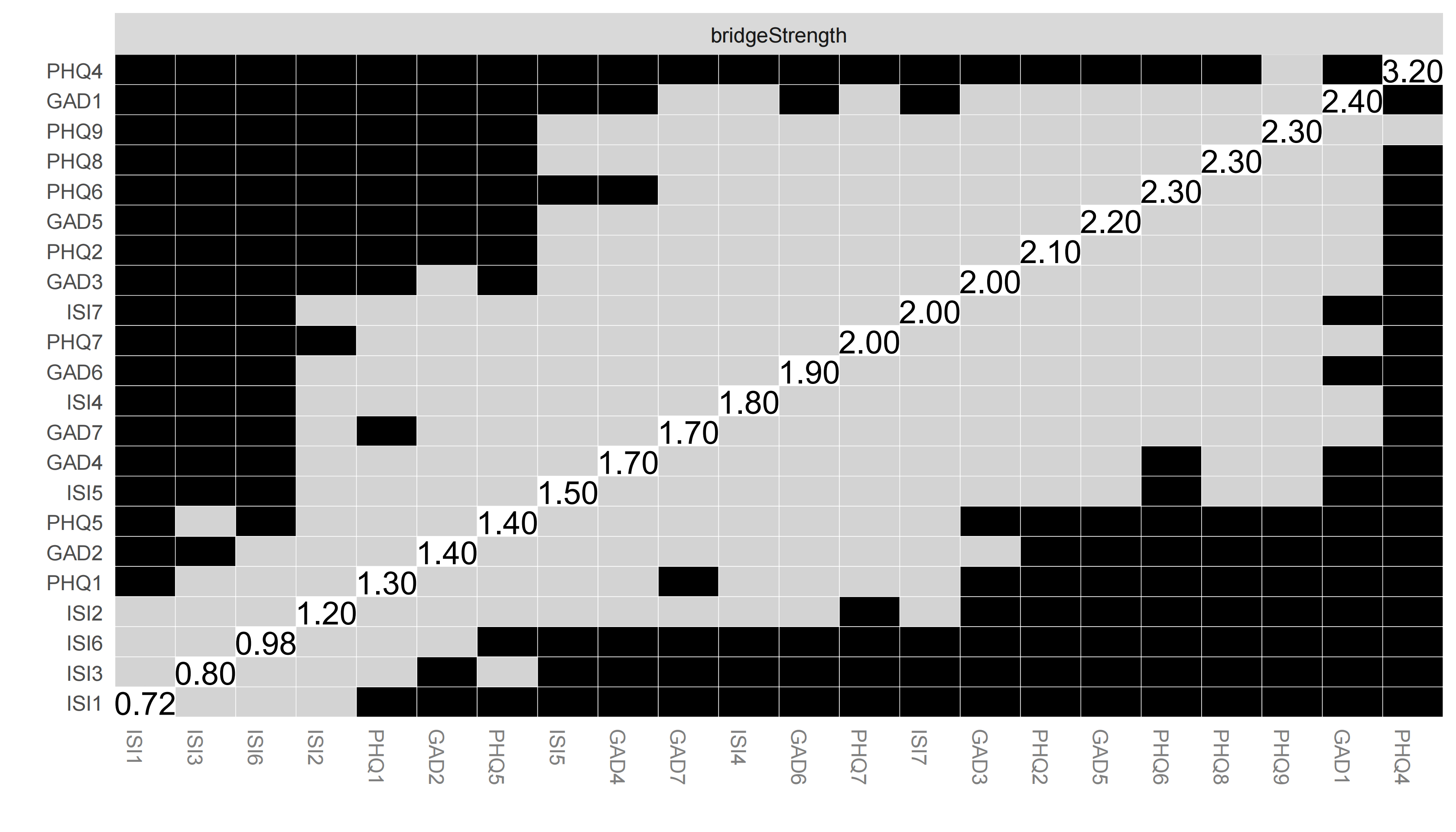

Supplement: Supplementary Figure 3 — Bootstrapped stability test for ‘bridge strength’. Bootstrapped difference tests between bridge strength of insomnia, anxiety, and depressive symptoms. Gray boxes indicate nodes that do not significantly differ from one another. Black boxes indicate nodes that differ significantly from one another (α = 0.05). White boxes show the values of bridge strength. [file Image3.tif]

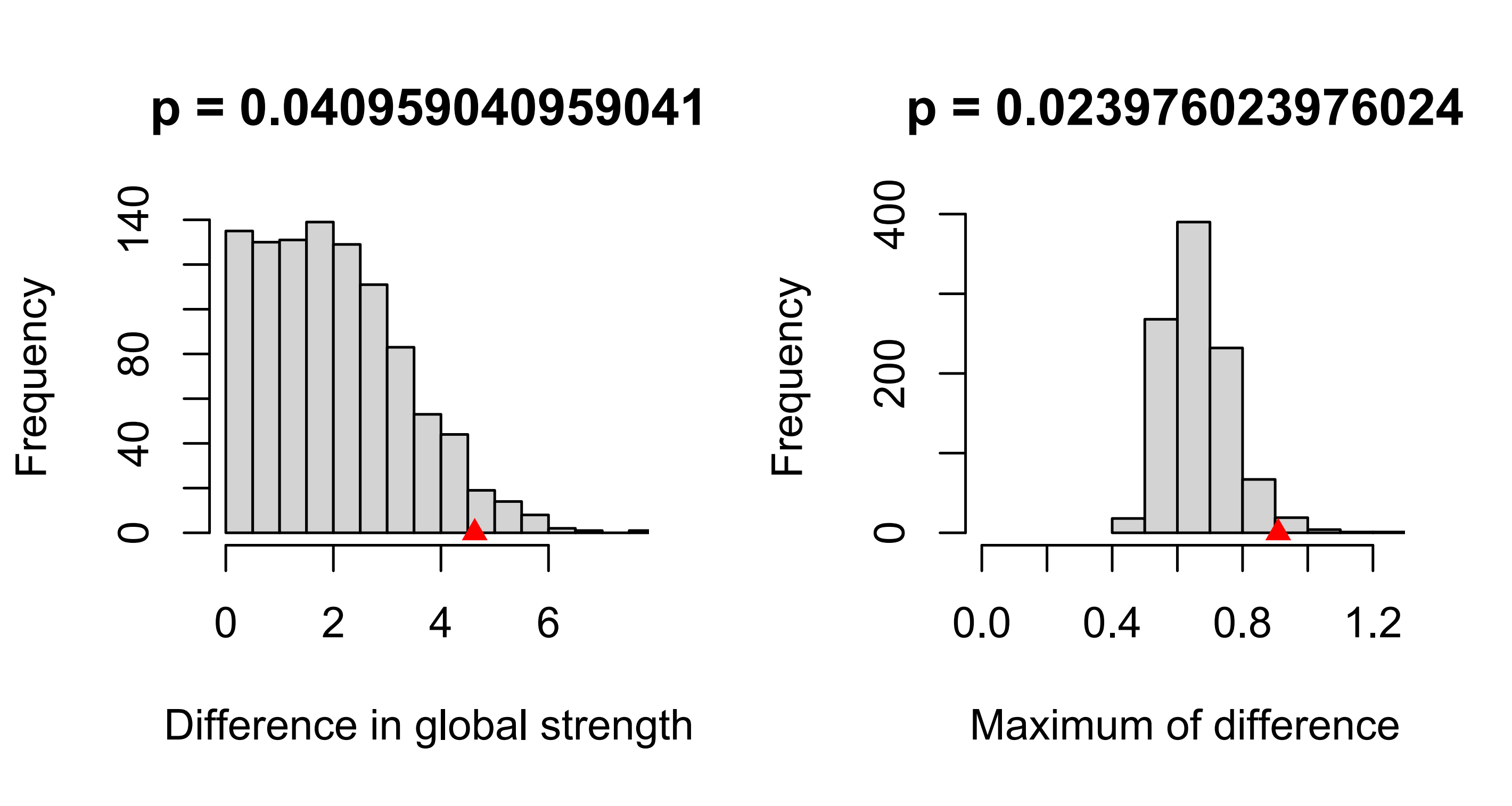

Supplement: Supplementary Figure 4 — Comparison of network properties between females and males. There was a significant difference in network global strength between females and males (Female: 70.527 vs. Male: 75.155; S = 4.628, p = 0.041), as well as in network structural invariance (M = 0.909, p = 0.025). [file Image4.tif]

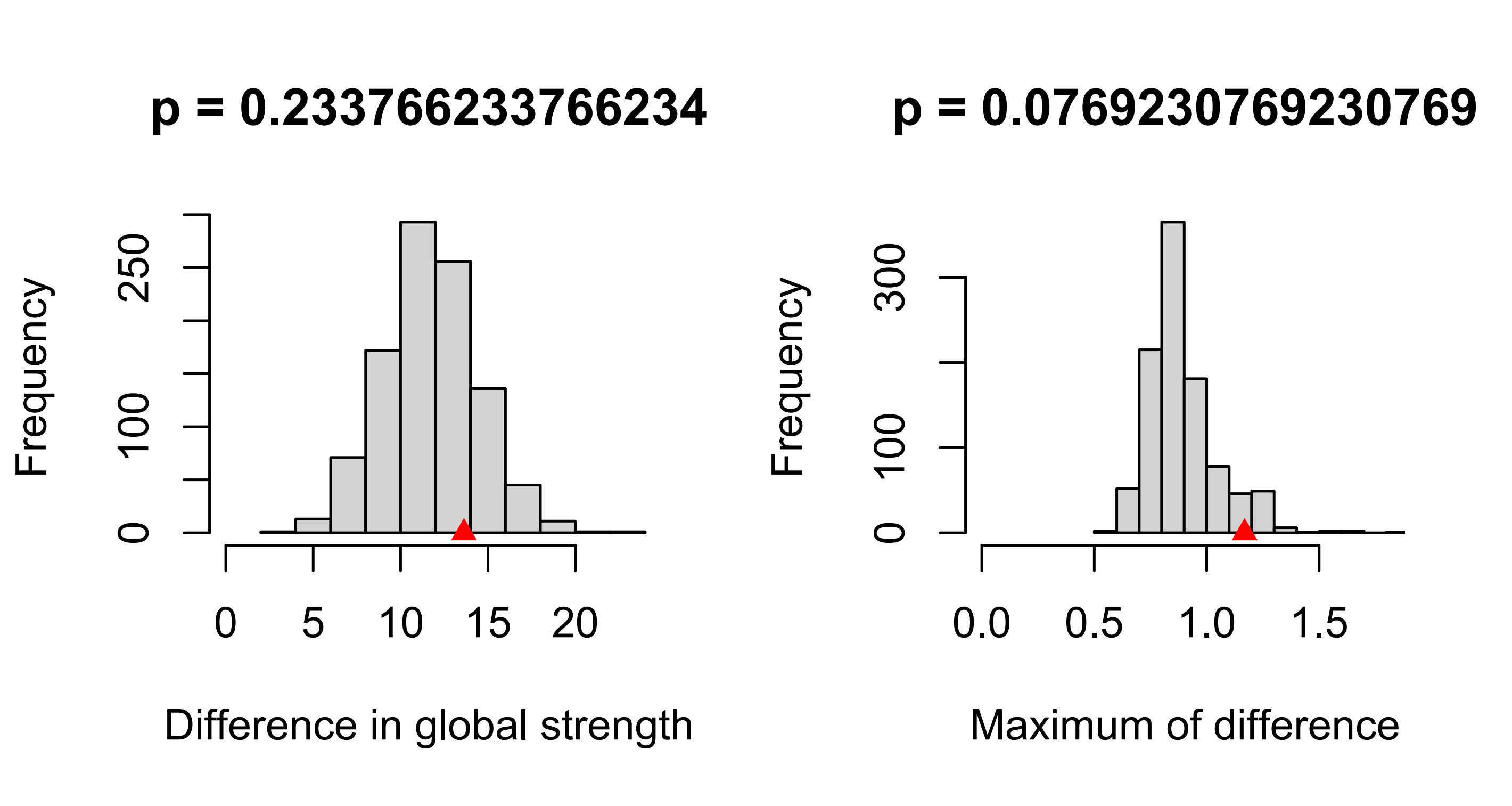

Supplement: Supplementary Figure 5 — Comparison of global strength and edge weights between quarantined and non-quarantined individuals. [file Image5.tif]

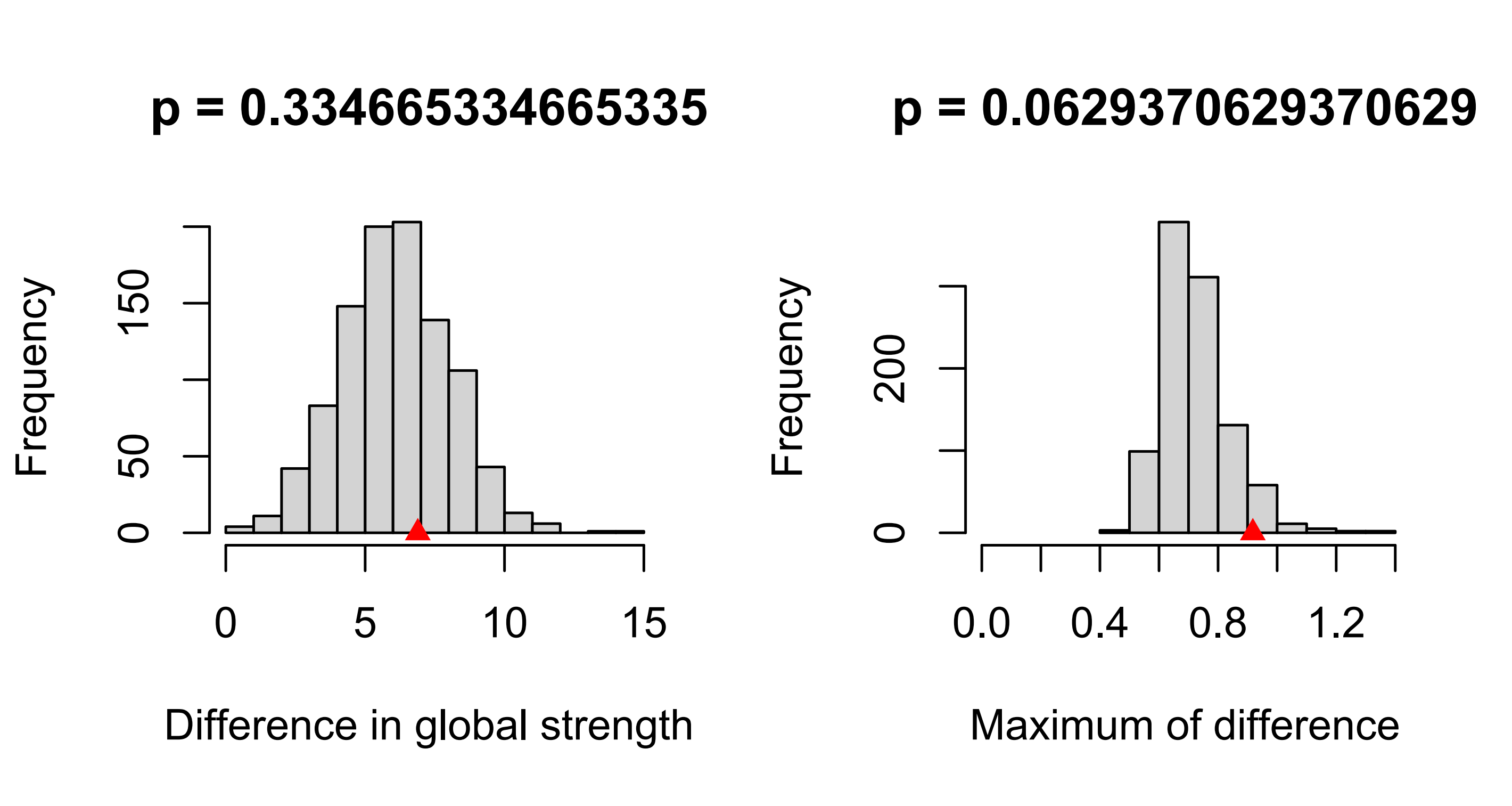

Supplement: Supplementary Figure 6 — Comparison of global strength and edge weights between groups with better and poorer family income. [file Image6.tif]
